# Assessment of change in limb length following correction of coronal and sagittal plane deformities in patients undergoing total knee arthroplasty: a retrospective study

**DOI:** 10.1186/s43019-026-00334-4

**Published:** 2026-07-23

**Authors:** Soutrik Kundu, Hyuck Min Kwon, Byung-Woo Cho, Jun Young Park, Woo-Suk Lee, Kwan Kyu Park

**Affiliations:** Department of Orthopedic Surgery, Yonsei University College of Medicin, Seoul, Korea, Republic of

**Keywords:** Limb length discrepancy, Total knee arthroplasty, Coronal plane deformity, Flexion contracture

## Abstract

**Background:**

Limb length discrepancy (LLD) following total knee arthroplasty (TKA) may affect functional outcomes and patient satisfaction. However, relative impact of coronal and sagittal plane deformity correction on change in limb length (CLL) following TKA are still not fully understood. The aim of this study is to guide the surgeon regarding the expected change in limb lengths following correction of coronal and sagittal plane deformities.

**Methods:**

This retrospective study comprised 237 primary TKAs conducted by a single surgeon from August 2024 to July 2025. Preoperative deformities consisted of 215 varus and 22 valgus knees. Standing anteroposterior radiographs of full length were used to assess the hip–knee–ankle (HKA) angle and limb length before and after the operation. Flexion contracture was assessed through clinical measurement. Correlation and multivariable regression analyses were conducted to ascertain predictors of limb length alteration, while adjusting for possible confounders such as patient height and polyethylene insert thickness. A regression-based predictive model was subsequently developed.

**Results:**

Mean limb length showed a notable increase after TKA (10.4 ± 9.1 mm; *p* < 0.0001). The preoperative HKA angle showed a significant positive correlation with CLL in both varus and valgus knees and remained the strongest independent predictor (*p* < 0.001 for varus; *p* = 0.046 for valgus). Preoperative flexion contracture did not show a significant independent association with CLL. Patient height and the thickness of the polyethylene insert did not have a notable effect on changes in limb length.

**Conclusions:**

TKA leads to consistent limb lengthening, mainly caused by the correction of coronal plane deformities. Estimating limb length changes before surgery can aid in surgical planning and patient counseling, especially in cases of unilateral or staged bilateral TKA.

*Level of evidence* III, Retrospective comparative cohort study

## Introduction

Total knee arthroplasty (TKA) effectively relieves pain and restores function in advanced knee arthritis, but outcomes are often influenced by preexisting deformities. Most of the patients undergoing TKA owing to osteoarthritis of the knee have a varying degree of varus deformity, often accompanied by a flexion contracture [1, 2]. On the contrary, inflammatory arthritis such as Rheumatoid Arthritis is usually associated with valgus deformity [3, 4].

Despite advancements in ERAS (Enhanced Recovery After Surgery) protocols enabling accelerated recovery, almost one out of five patients undergoing TKA are not fully satisfied by the results of the surgery [5–9] and LLD can be a significant contributor to such discontentment [10–13]. LLD following total hip arthroplasty (THA) is well documented, occurring in around 30% of patients and reaching up to 17 mm, and is a significant cause of dissatisfaction [14, 15]. Several studies have suggested changes in limb length following TKA which are significantly correlated with coronal plane deformity but not with sagittal plane deformity [16, 17]. Three-dimensional simulation studies identified femoral and tibial dimensions as contributing factors to postoperative changes in limb length; however, separate measurement of these bones was considered impractical [18]. This limitation may be addressed by incorporating patient height into the analysis, given that femoral and tibial lengths are expected to vary proportionally with stature [19]. Khalifa et al. suggested that, a medically fit patient with bilateral knee arthritis associated with severe deformity, concurrent bilateral surgery should be advocated to avoid LLD following unilateral surgery [20] as discrepancies more than 20 mm can cause significant discomfort [21, 22].

The aim of our study is to guide the surgeon regarding the expected change in limb lengths following correction of coronal and sagittal plane deformities for patients of varying height, so that an informed decision can be made regarding the treatment plan and enhance functional outcome of the patients following surgery.

## Methods

### Study design

This retrospective record-based study was conducted in the department of orthopedic surgery at a tertiary teaching hospital in accordance with STROBE guidelines after receiving approval from the Institutional Review Board (IRB No.-4-2025-1048). Data extraction was carried out in compliance with study ethics guidelines. Informed consent was not necessary because this was a retrospective study and all patient data were de-identified prior to analysis.

Operative data for all patients who underwent elective primary TKA from August 2024 to July 2025, by a single senior surgeon were retrieved from hospital records. All the surgeries were done using principles of mechanical alignment with a measured resection technique, thereby minimizing variability.

### Inclusion and exclusion criteria

**Fig. 1:**
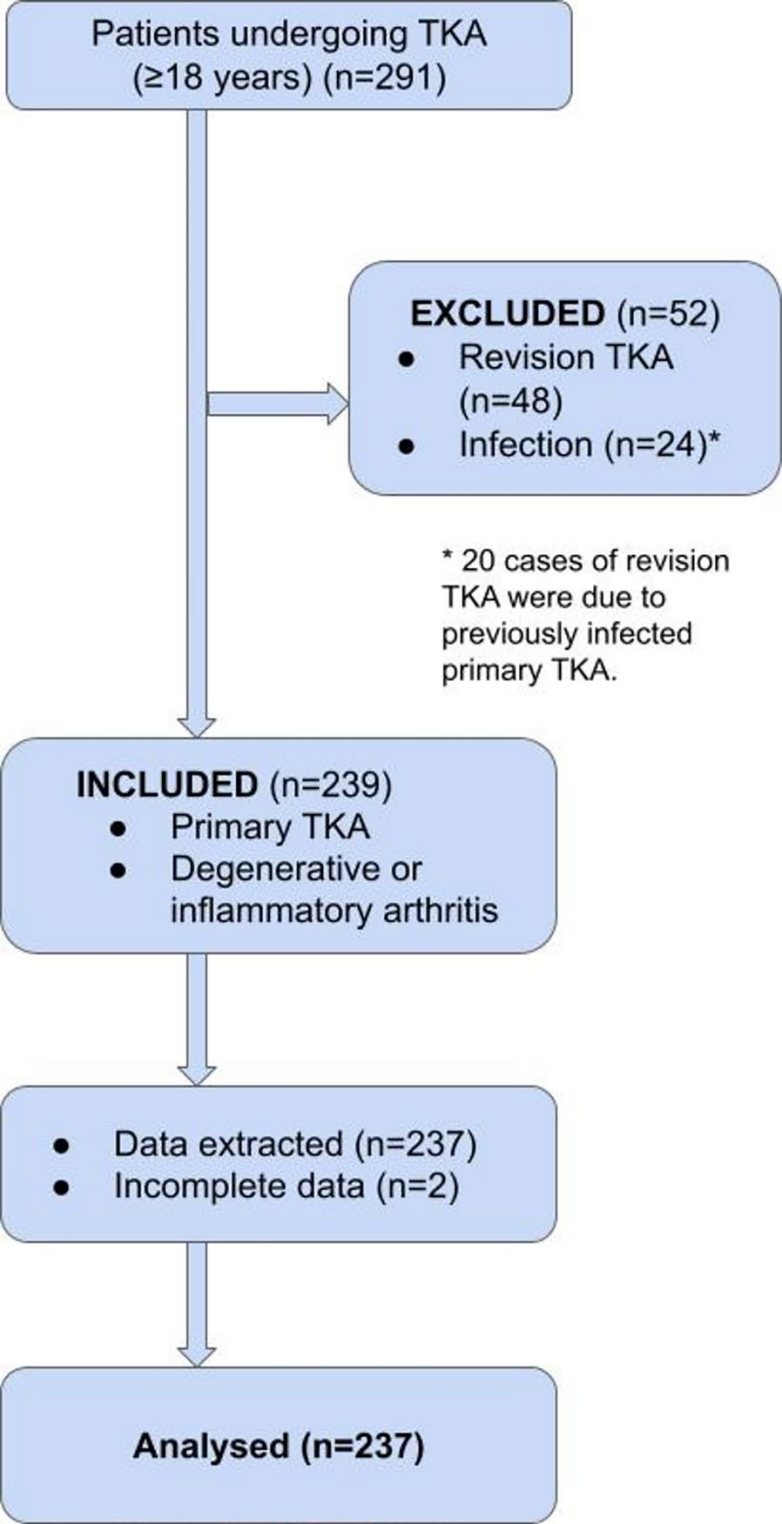
Flowchart illustrating patient selection

The study included patients aged over 18 years who underwent elective primary TKA for degenerative or inflammatory arthritis. Patients who underwent revision arthroplasty, arthroplasty for infective etiologies, or those with incomplete data were excluded from the study (Fig. 1).

### Data extraction

Relevant patient data—including age, sex, weight, height, date of surgery, preoperative and postoperative hip-knee-ankle (HKA) angle, preoperative flexion contracture (FC), poly insert size, and preoperative and postoperative limb lengths—were extracted from electronic medical records (EMR).

**Fig. 2:**
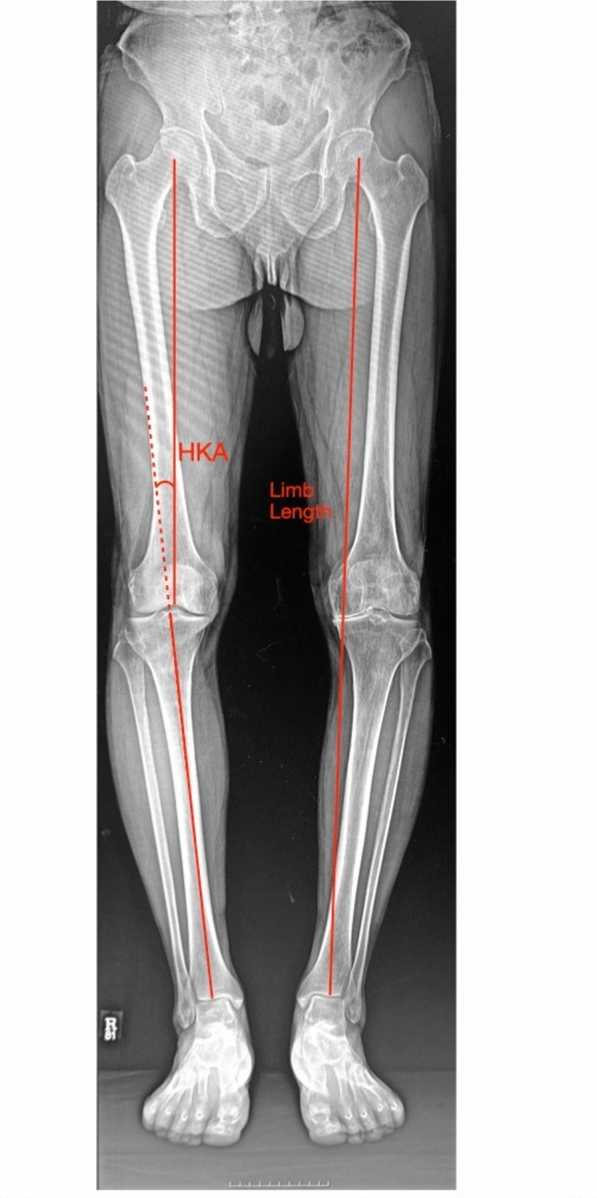
Assessment of hip-knee-ankle angle and limb length

The HKA angle and limb lengths were measured using preoperative and postoperative full length standing anteroposterior (AP) radiographs (Fig. 2). HKA angle was expressed in terms of deviation from neutral mechanical alignment (0°) with varus and valgus expressed as directional angular deviation rather than absolute angle values. Postoperative radiographic assessment was performed at 1-month follow-up and the patients were subsequently followed up for a duration of 6–18 months (median = 11.8 months). The patient was asked to stand erect with their bare feet placed close together and knee extended as far as possible. Change in limb length (CLL) was calculated as the difference between preoperative and postoperative limb length readings. FC was measured clinically using a handheld goniometer as part of the preoperative clinical assessment performed 1 day prior to surgery. Data collection was done by an independent, blinded researcher not involved in patient care or surgery.

### Statistical analysis

Continuous data was represented as mean ± standard deviation (SD). Categorical data were represented as proportions (percentages).

Preoperative and postoperative limb lengths satisfied the assumption of normality across all datasets, as assessed using the Shapiro–Wilk test. Accordingly, the mean and standard deviation (mean ± SD) were reported for each of these variables and were compared using the paired *t*-test.

Partial Spearman correlation analysis was performed to assess the relationship between change in limb length (CLL) and preoperative measures (HKA angle and FC) after adjusting for potential confounders (patient height and polyethylene insert size). A *p*-value < 0.05 was considered statistically significant. Univariable analyses assessed the one-to-one association between Change in limb length and each predictor. To develop a predictive model for limb length change, relevant variables were entered into a multiple regression analysis, and a visual matrix was generated on the basis of the regression output. Variance inflation factor (VIF) was used to assess multicollinearity among the independent variables in the multivariable model. Statistical analysis was done using R package, version 4.4.2 (http://www.R-project.org).

## Results

A total of 237 primary TKA cases were analyzed, consisting of 215 patients with preoperative varus deformity and 22 with valgus deformity. The maximum preoperative deformities observed were 24 degrees varus and 22 degrees valgus, with flexion contracture ranging up to 30 degrees. There was only one patient with 5 degrees recurvatum deformity, who had previously undergone medial open wedge high tibial osteotomy and was excluded from this analysis.

The mean preoperative varus deformity was 11 degrees, while the mean valgus deformity was 6.2 degrees. Postoperatively, the mean residual deformity was 1.5 degrees of varus in patients with preoperative varus deformity and near-neutral alignment in those with preoperative valgus deformity. Detailed patient characteristics are presented in Table 1.

**Table 1:** Patient characteristics

	Varus (*n* = 215)	Valgus (*n* = 22)	Total (*n* = 237)
Age	73.0 ± 5.2	71.5 ± 9.0	72.9 ± 5.7
Sex (M/F)	30/185	5/17	35/252
Side (R/L)	106/109	10/12	116/121
Height	155.9 ± 6.7	159.2 ± 7.5	156.2 ± 6.9
BMI	26.6 ± 4.0	26.3 ± 3.6	26.6 ± 4.0
Deformity			
Preoperative HKA	11.0 ± 4.8	6.2 ± 5.6	NA
Postoperative HKA	1.5 ± 2.3	−0.1 ± 2.7	NA
Preoperative flexion contracture	7.3 ± 5.7	6.6 ± 6.6	7.3 ± 5.7
Change in limb length (mm)	10.1 ± 8.9	12.5 ± 10.9	10.4 ± 9.1

Descriptive statistics for continuous variables are presented as mean ± SD. Categorical variables were summarized as counts for each category. Negative HKA signifies valgus alignment

Table 2 compares preoperative and postoperative limb lengths, demonstrating a significant increase following surgery in varus, valgus, and overall cohorts (*p* < 0.0001).

**Table 2:** Changes in limb length between pre- and postoperative measurements

	Varus	Valgus	Total
Preoperative limb length	77.7 ± 4.9	78.9 ± 5.6	77.8 ± 5.0
Postoperative limb length	78.8 ± 4.9	80.1 ± 5.8	79.0 ± 5.0
*p*-Value	< 0.0001	< 0.0001	< 0.0001

Data has been represented as mean ± standard deviation (SD)

**Fig. 3:**
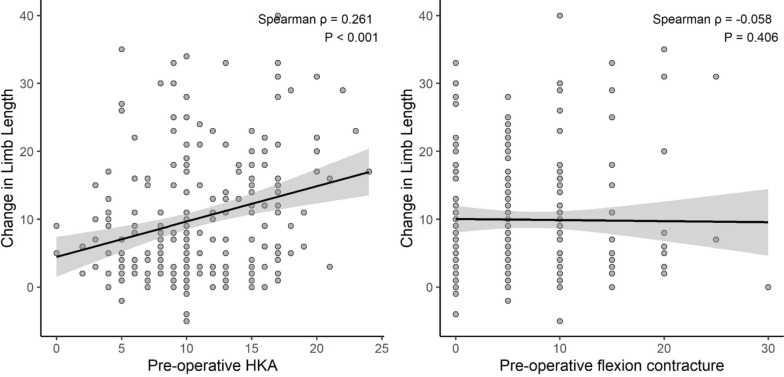
Scatterplot for varus knees undergoing TKA

**Fig. 4:**
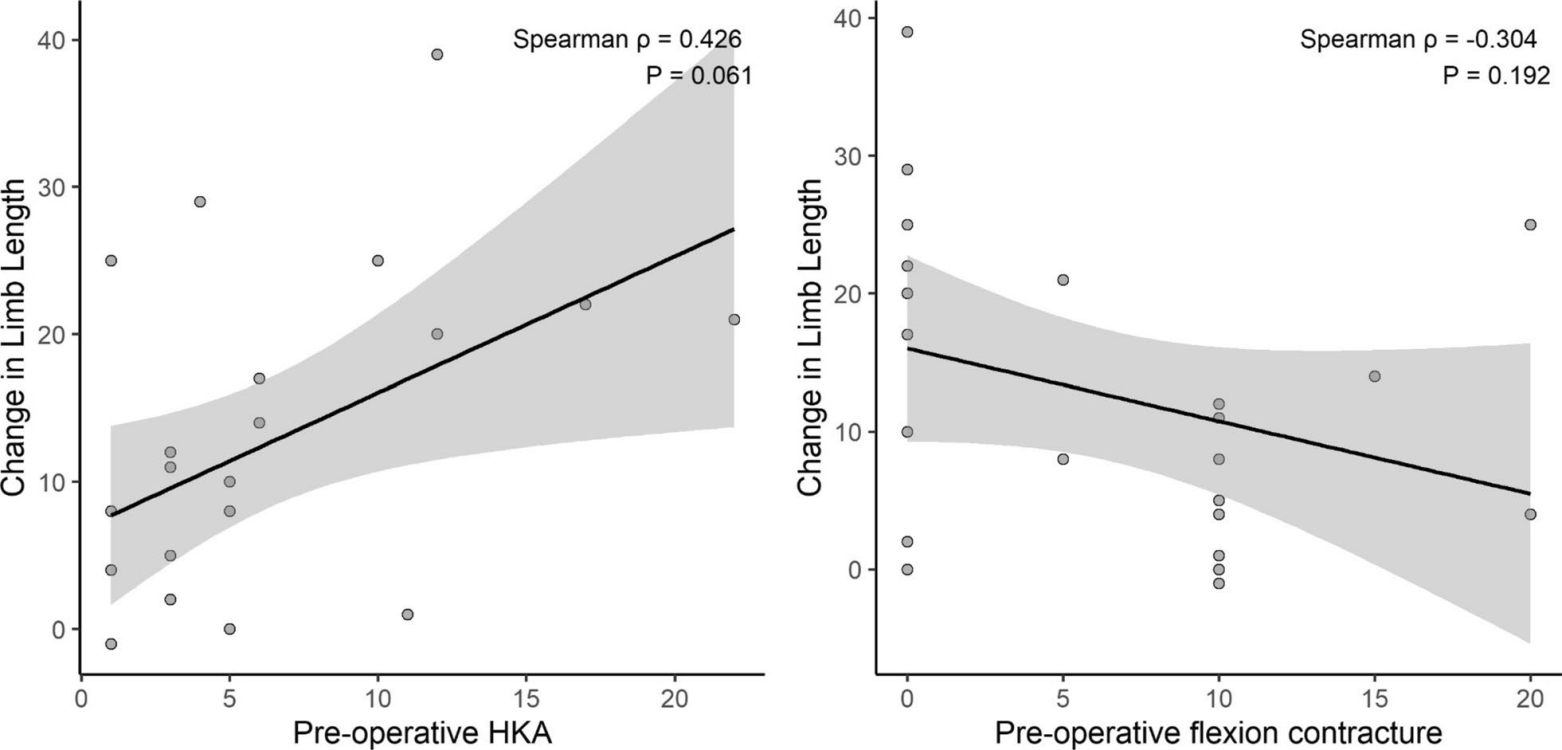
Scatterplot for valgus knees undergoing TKA

A significant positive correlation was observed between CLL and preoperative HKA angle in varus knees. However, no significant correlations were found between preoperative FC and CLL in varus knees, nor between preoperative HKA angle or FC and CLL in valgus knees (Figs. 3, 4 and Table 3).

**Table 3:** Partial correlation between change in limb length and preoperative measures (results are adjusted for height and insert size)

Variable	Varus		Valgus	
	$\rho$(95% CI)	*p*-Value	$\rho$(95% CI)	*p*-Value
Preoperative HKA angle	0.26 (0.13, 0.38)	0.0002	0.43 (−0.02, 0.73)	0.0614
Preoperative FC	−0.06 (−0.19, 0.08)	0.4065	−0.30 (−0.66, 0.16)	0.1925

Preoperative HKA angle showed a significant positive association with Change in limb length in both varus (*p* < 0.0001) and valgus knees (*p* = 0.0263). Height and polyethylene insert size were not significant predictors and were therefore excluded from the final multivariable model. Preoperative HKA remained a significant determinant of Change (*p* < 0.0001 for varus; *p* = 0.0461 for valgus) in limb length after adjustment for patient height and insert size (Table 4 and 5).

**Table 4:** Linear regression analysis of change in limb length (varus)

	Univariable		Multivariable	
	$\beta$(SE)	*p*-Value	$\beta$(SE)	*p*-Value
Preoperative HKA angle	0.521 (0.125)	< 0.0001	0.542(0.122)	< 0.0001
Preoperative FC	−0.016 (0.107)	0.8831	−0.061 (0.103)	0.5516
Height	0.003 (0.090)	0.9747		
Polyethylene insert size	0.306 (0.708)	0.6657

**Table 5:** Linear regression analysis of change in limb length (valgus)

	Univariable		Multivariable	
	$\beta$(SE)	*p*-Value	$\beta$(SE)	*p*-Value
Preoperative HKA angle	0.925 (0.385)	0.0263	0.883 (0.390)	0.0461
Preoperative FC	−0.527 (0.351)	0.1488	−0.384 (0.330)	0.2595
Height	0.427 (0.313)	0.1871		
Polyethylene insert size	5.695 (4.973)	0.2656		

Therefore predictive equations to assess Change in Limb Length (CLL) are:

CLL (varus) = 4.441 + 0.553*HKA—0.061*FC, and

CLL (valgus) = 9.889 + 0.833*HKA—0.384*FC,

Where HKA and FC signify preoperative HKA angle and preoperative flexion contracture respectively. The result will be in millimeters. From the above equation we have generated a visual matrix (Table 6).

**Table 6:** Predictive matrix to determine change in limb length from preoperative hip-knee-ankle (HKA) angle and flexion contracture

		Preoperative HKA angle								
		Valgus					Varus			
Preoperative flexion contracture		**20**	**15**	**10**	**5**	**0**	**5**	**10**	**15**	**20**
	0	26.5	22.4	18.2	14.1	4.4	7.2	10.0	12.7	15.5
	5	24.6	20.5	16.3	12.1	4.1	6.9	9.7	12.4	15.2
	10	22.7	18.5	14.4	10.2	3.8	6.6	9.4	12.1	14.9
	15	20.8	16.6	12.5	8.3	3.5	6.3	9.1	11.8	14.6
	20	18.9	14.7	10.5	6.4	3.2	6.0	8.8	11.5	14.3
	25	–	–	–	–	2.9	5.7	8.4	11.2	14.0
	30	–	–	–	–	2.6	5.4	8.1	10.9	13.7

Maximum data range for flexion contracture for valgus knees in our study was 20 degrees. Therefore data beyond this range cannot be used for prediction

## Discussion

In this study we have found that TKA is associated with a consistent and statistically significant increase in limb length. The most important finding is that the preoperative coronal plane deformity (HKA angle) remains the primary determinant of change in limb length.

The strong positive association between preoperative HKA angle and postoperative change in limb length observed in this study aligns with the existing evidence by Chalmers et al., Hinarejos et al. and Marmor et al. [10, 12, 16] Biomechanically, restoration of the mechanical axis causes an increase in the effective hip-to-ankle distance by converting the angular deformity into linear gain, a principle well described in deformity correction theory. This explains the linear relationship observed in both varus and valgus knees, with greater deformity correction resulting in greater limb lengthening. This study further demonstrated that this finding remained significant even after adjusting for potential confounders such as patient height and polyethylene insert thickness. While this relationship is well established in existing literature, the predictive matrix derived from this model may serve as a practical tool for preoperative estimation of limb length change in routine clinical practice.

It is worthy to note that valgus knees showed a numerically greater mean increase consistent with findings in prior studies [16]. This results possibly due to lateral femoral condylar hypoplasia, lateral soft-tissue contractures, and the need for more extensive releases. However, it is important to note that the smaller number of valgus knees in the present cohort limits its statistical power and caution should be exercised while extrapolating predictions for extreme valgus knees.

Contrary to coronal plane deformity, preoperative flexion contracture was not significantly associated with postoperative change in limb length. This finding is consistent with previous studies, including those by Chang et al., which reported minimal influence of sagittal plane deformity on radiographic limb length following TKA [17]. Flexion correction is primarily achieved through soft-tissue release and posterior capsular balancing rather than bony alterations that modify overall limb geometry [23], which may explain the lack of significant correlation. Therefore, the hip-to-ankle distance evaluated on standing radiographs may not be significantly altered by restoration of extension.

However, this observation should be interpreted with caution. Anteroposterior radiographs taken in a standing position are sensitive to knee flexion, and the presence of flexion contracture may result in a seemingly reduced limb length attributable to projectional variables, such as the parallax effect. Consequently, the restoration of complete extension in the postoperative phase may manifest as a perceived augmentation in measured limb length, despite the absence of any genuine geometric alteration. It is important to note that, this reestablishment of extension might be interpreted by the patient as an increase in limb length, even in the lack of structural modification, thereby underscoring the critical differentiation between radiographically assessed and perceived limb length, as well as its potential implications for postoperative functional rehabilitation.

Despite serving as a surrogate for femoral and tibial dimensions, patient height did not significantly predict alterations in limb length. This suggests that variations in postoperative limb length are predominantly governed by angular correction as opposed to the absolute length of the bone, a finding consistent with prior biomechanical research. Likewise, the thickness of the polyethylene insert exhibited no independent influence on the modification of limb length; this may be attributed to the fact that insert thickness affects joint line elevation rather than the overall length of the limb.

## Limitations

This study is subject to several limitations. The retrospective nature of its design introduces an inherent selection bias, and given that all procedures were conducted by a single surgeon utilizing a mechanical alignment technique, the findings may not be applicable to kinematic alignment, robotic-assisted total knee arthroplasty, or other surgical techniques. Furthermore, the relatively limited sample size of valgus knees constrains the statistical power and compromises the reliability of conclusions drawn from subgroup analyses, particularly in instances of significant deformity. Additionally, important intraoperative determinants, such as the amount of bone loss, resection depth, and extent of soft tissue release, could not be quantitatively assessed owing to the retrospective nature of the study and could have influenced the postoperative limb length, potentially confounding the observed association between deformity correction and limb lengthening. The relatively short enrollment period may also limit the generalizability of the findings, and larger longitudinal studies are warranted.

This study utilized standing full-length anteroposterior radiographs, which, despite their prevalent application and clinical utility, yield a two-dimensional evaluation and do not account for rotational alignment or intricate three-dimensional deformities. Moreover, the exclusion of patient-reported outcome measures (PROMs) constrains the capacity to ascertain the clinical relevance of the detected alterations in limb length. The radiographic assessment of limb length discrepancy may not necessarily align with patient experiences, adaptive gait mechanisms, or functional results, particularly in the aftermath of deformity correction and the reestablishment of extension. Subsequent research endeavors that integrate three-dimensional imaging, patient-reported outcome measures, and gait analysis are imperative to more accurately delineate clinically significant thresholds of limb length variation.

## Conclusions

Total knee arthroplasty results in a predictable increase in limb length, which appears to be primarily influenced by the correction of coronal plane deformities. Preoperative hip-knee-ankle (HKA) angle was the most significant predictor of alterations in limb length and can be used to forecast postoperative changes in limb length through a straightforward predictive model. Preoperative flexion contracture did not exhibit a significant independent association with radiographic changes in limb length and should be interpreted within the context of measurement constraints and subjective patient perceptions. These observations may assist in preoperative planning and patient counseling, especially in cases involving unilateral or staged bilateral total knee arthroplasty. Future prospective studies that integrate three-dimensional evaluations, gait analysis, and patient-reported outcomes are necessary to establish clinically meaningful thresholds.

## Data Availability

No datasets were generated or analyzed during the current study.
